# Hearing technology and the brain: cognitive function in different hearing device users

**DOI:** 10.3389/fnagi.2026.1887207

**Published:** 2026-08-05

**Authors:** Bamini Gopinath, Jessica Turner, George Burlutsky, Shermin Lim, Alicia Zou, Ghazal Shineh, Kerry Sherman, Kompal Sinha, Diana Tang

**Affiliations:** 1 Macquarie University Hearing, Department of Health Sciences, Macquarie University, Sydney, NSW, Australia; 2 Macquarie University Hearing Research Centre, Macquarie University, Sydney, NSW, Australia; 3 College of Education, Psychology & Social Work, Flinders University, Adelaide, SA, Australia; 4 Lifespan Health and Wellbeing Research Centre, Macquarie University, Sydney, NSW, Australia; 5 School of Psychological Sciences, Macquarie University, Sydney, NSW, Australia; 6 Macquarie Business School, Department of Economics, Macquarie University, Sydney, NSW, Australia; 7 Macquarie University Health at Work Research Center, Macquarie University, Sydney, NSW, Australia

**Keywords:** adults, cognitive function, epidemiology, hearing devices, hearing loss

## Abstract

**Background:**

There is an evidence-gap on how different hearing devices influence cognition. This study aimed to simultaneously compare cognitive performance across key domains among hearing aid, cochlear implant and bimodal device users.

**Methods:**

Cross-sectional data were collected and analyzed as part of the Hearing Impairment in Adults: A Longitudinal Outcomes Study, during 2022–24. Adults aged ≥40 years old using hearing aids, cochlear implants, or bimodal hearing devices were recruited. Of 1054 approached, 617 participants had complete data: 387, 127, and 103 hearing aid, cochlear implant and bimodal device users, respectively. Cognitive function was assessed using the Cogstate Brief Battery among the different device user groups.

**Results:**

In the overall population, no significant differences were observed in Cogstate Brief Battery scores between device users. Age-stratified analyses (<70 and ≥70 years) was conducted, and cochlear implant compared to hearing aid users aged <70 had better reaction times in the following test – Detection (psychomotor function): 2.54 versus 2.58 (*p* = 0.02). In participants aged ≥70, cochlear implant users compared to hearing aid users showed poorer visual attention or higher mean Identification test scores: 2.83 versus 2.78 (*p* = 0.02).

**Conclusion:**

These findings suggest that hearing device type is not uniformly associated with cognitive performance, but rather that age and cognitive domain critically shape observed differences. This underscores the importance of more targeted cognitive monitoring in adults with hearing loss.

## Introduction

Hearing loss is a prevalent chronic condition and one of the top three leading causes of disability, globally (Haile et al., 2024). If unaddressed, hearing loss can have a negative impact on participation in social activities, mental health, functional independence, aging, and overall lifespan (Gopinath et al., 2009, 2012, 2021; Prieur Chaintré et al., 2024). Midlife hearing loss was identified as one of the largest modifiable risk factors for a future dementia diagnosis (Livingston et al., 2024). While the exact mechanisms by which hearing loss influences cognition in adults is not established, hypothesized pathways include hearing loss increasing cognitive load, reducing sensory input, and/or limiting social engagement, all of which can accelerate cognitive decline (Fulton et al., 2015; Griffiths et al., 2020; Livingston et al., 2024).

Addressing hearing loss may prevent up to 9% of dementia cases worldwide (Livingston et al., 2024). Hearing aids are recommended for mild to moderate hearing loss while cochlear implants are recommended for severe to profound hearing loss where hearing aids are insufficient. The effects of hearing aid and/or cochlear implant use on cognitive performance and dementia risk remain unclear. To date, only one randomized large-scale clinical trial of hearing aids has been conducted, this reported no benefit of device use on cognitive outcomes after 3 years, although a sub-group at greater risk of cognitive decline showed a near 50% reduction in the rate of decline after hearing intervention (Lin et al., 2023). Nevertheless, our recent umbrella review (Tang et al., 2025) concluded that the effects of hearing aid use on cognition are unclear, with more null than positive findings and substantial methodological limitations across studies. Regarding the literature on cochlear implantation and dementia, a large nationwide cohort study in Korea recently reported that cochlear implant recipients consistently showed a lower proportion of new dementia diagnoses compared with non-recipients, indicating a significant association between cochlear implant use and a reduced risk of dementia (Chang et al., 2025). Our umbrella review (Tang et al., 2024) also revealed that there are improvements or maintenance of cognitive outcomes in cochlear implant users after 1-year, however, more robust studies are needed to determine the impact on cognition especially as the quality of current primary research is uncertain due to e.g., small sample size (Tang et al., 2024).

As noted in previous research (Sarant et al., 2023), a significant methodological limitation across many studies is the reliance on inappropriate cognitive assessment methods, including insensitive cognitive screening tools and the administration of tests to individuals with hearing loss using auditory instructions. Other methodological issues include small sample size, reliance on self-reported data or absence of objective hearing loss assessment, inadequate adjustment for confounding variables, and the absence of a comparative or control group (Bucholc et al., 2021; Deal et al., 2015). Additionally, while cohort studies have assessed cognitive function in hearing aid and cochlear implant users separately (Sarant et al., 2023, 2024), no study to date has compared differences in cognitive function across all three device groups – hearing aid, cochlear implant and bimodal device (cochlear implant and hearing aid) users.

Our observational study of adults aged 40+ years aims to address these evidence-gaps by simultaneously comparing cognitive function among hearing aid, cochlear implant and bimodal device users. To our knowledge, this study provides the first opportunity to evaluate whether different device types are associated with differences in cognitive performance across key domains, using a standardized, validated cognitive battery. These novel epidemiological data can contribute clinically actionable evidence to guide hearing intervention strategies focused not only on improving communication, but also in supporting long-term brain health in adults at risk of cognitive decline in later life.

## Methods

### Participants and recruitment

HALOS (Tang et al., 2023) is an ongoing mixed-methods, prospective cohort study designed to assess the effects of hearing device use on a broad range of health and social outcome measures. The protocol for this study has been published (Tang et al., 2023). Briefly, the present study focuses on cross-sectional analysis of baseline (wave 1) data collected during 2022–24. Participant recruitment was conducted through flyers distributed by hearing service providers across the country and social media advertisements (e.g., Facebook). Interested individuals contacted the research team by phone or email to express their interest in participating in the study. Study inclusion criteria were individuals aged 40+ years who use cochlear implants and/or hearing aids in at least one ear; and have adequate English proficiency to complete the questionnaires.

All participants provided written informed consent, and the study received ethical approval from Macquarie University Human Research Ethics Committee (ID: 11262) and Southern Adelaide Clinical Human Research Ethics Committee (LNR/22/SAC/88). The study was registered with the Australia New Zealand Clinical Trial Registry (ACTRN12622000752763).

### Data collection

Participants self-completed an online survey via Research Electronic Data Capture (REDCap) or paper-based survey via post/email within a 2-week period. Participants also self-completed the computerized Cogstate Brief Battery (CBB). Participants on average took 75 min to complete all aspects of the study.

### Study factors

Participants were categorized based on self-reported hearing device usage, including hearing aid users, cochlear implant users, and bimodal users. Additional covariates considered in the analysis were age, sex, education level, and duration of hearing device use (years since receiving the current device). Hearing function was evaluated using the two validated self-report instruments: the Attitude toward loss of Hearing Questionnaire (ALHQ), which assesses hearing-related self-efficacy, stigma, and coping behaviors across multiple subscales (including vision/dexterity), providing insights into psychosocial adjustment to hearing loss; and the Speech, Spatial, and Qualities of Hearing Scale (SSQ), a 12-item tool that captures self-reported hearing ability in complex listening environments. Health-related quality of life was measured using the Health Utilities Index 3 (HUI3), a preference-based instrument that generates utility scores from multiple health domains, including hearing, vision, and mobility (Horsman et al., 2003).

The most recent audiometric data was obtained for all participants through their authorized consent to release this information from their hearing service provider. These data included audiometric thresholds for air conduction stimuli in both ears for octave frequencies at 0.25–8.0 kHz and/or four-frequency average hearing loss, measured at 0.5, 1, 2 and 4 kHz. Hearing loss was determined as the four-frequency pure-tone average of audiometric hearing thresholds at 500, 1000, 2000 and 4000Hz. Hearing loss was stratified as mild (>25–40, decibels hearing level, dB HL) and moderate or greater hearing loss (>40 dB HL).

### Outcome measures

Cognition was assessed using the CBB. The CBB has been optimized for use in unsupervised contexts (Banh et al., 2022; Lim et al., 2012), and with high sensitivity to small changes in cognitive performance and longitudinal decline in older adults, high reliability and specificity for different cognitive functions, and visual (non-auditory) presentation which makes this tool highly suitable for use with people with hearing loss (Sarant et al., 2023, 2024). The CBB consists of the Detection test (Detection) – a test of simple reaction time that measures psychomotor function; the Identification test (Identification) – a choice reaction time test that measures visual attention; the One Card Learning test – a visual recognition test that measures visual learning; and the One-Back test – an n-back test that measures working memory. The CBB has been described in detail, previously (Lim et al., 2012). Technical support was provided by the study team where required to help run the assessments, but no assistance was provided around how to complete the tasks, such as explaining the instructions to them. There is also a practice run for each task. If they fail the practice they do not proceed to the assessment component, and it moves on to the next task. At the end of the four tasks, they will return to the task that they failed the practice to try again. Results were screened in accordance with Cogstate’s established completion and integrity checks.

Responses to each test were recorded in terms of the mean reaction time to measure speed, and proportion of correct answers to measure accuracy. Accuracy was used as the primary outcome measure for One Card Learning and One-Back tests, with higher scores indicating better accuracy or cognitive performance in these tasks. Speed was used as the primary outcome for Detection and Identification; with lower scores indicating quicker reaction time or better cognitive performance in these tasks. For each participant, outcome scores on the CBB tests were expressed as standard scores using the mean and standard deviation of the entire sample.

### Statistical analysis

General Linear Models (PROC GLM) was used for all analyses. GLM provided one-way analysis of variance (ANOVA) and analysis of covariance (ANCOVA). To determine whether there were differences in study characteristics between the device groups, means and standard deviations were calculated by PROC MEANS and *p*-values were determined by ANOVA for continuous variables (e.g., age). For categorical variables (e.g., sex), *n* (%) was calculated by PROC FREQUENCY and *p*-values were determined by Chi-square tests.

ANCOVA was used to examine associations between hearing device type (hearing aids, cochlear implants, bimodal devices) and CBB scores i.e., psychomotor function (Detection), working memory (One-Back Card), visual attention (Identification), and visual learning (One Card Learning). Least squares means were calculated to compare adjusted group means, and *post hoc* pairwise comparisons were conducted with *p*-values presented, by first adjusting for age and sex, and then further adjusting for covariates that remained significant in the final multivariable model: education level (having or not having tertiary qualifications), duration of device usage (number of years used), HUI-3, SSQ, ALHQ vision/dexterity subscale scores and hearing loss severity (<40 dB HL or ≥40 dB HL). Other factors such as number of devices (unilateral or bilateral), hearing loss duration and several comorbidities (e.g., cardiovascular disease, diabetes) were assessed but were not significant covariates in the multivariate model and thus, were not adjusted for in final analysis. Effect sizes across the three hearing device user groups were estimated using Partial Eta-Squared.

A Bonferroni correction was applied to the ANCOVA results to account for multiple testing (Table 1). To control the family-wise error rate due to multiple comparisons, Bonferroni-adjusted *p*-values were used for all pairwise comparisons among groups. The analyses were performed using the Bonferroni option in SAS PROC GLM, which adjusts *p*-values for multiple pairwise differences following the ANCOVA. Analyses were conducted using SAS (v9.4), with significance set at *p* < 0.05.

**TABLE 1:** Study characteristics of device user groups.

Device user groups					
Characteristics	Hearing aid (*n* = 387)	Bimodal (*n* = 103)	Cochlear implant (*n* = 127)	Total (*n* = 617)	*P*-value
Age (years), Mean (SD)	71.7 (9.9)	66.4 (11.7)	62.0 (11.5)	68.8 (11.3)	<0.0001
Females, *n* (%)	229 (59.3)	53 (51.5)	75 (59.1)	357 (58.0)	0.3419
Tertiary education, *n* (%)	302 (78.0)	78 (75.7)	105 (82.7)	485 (78.6)	0.3998
SSQ, Mean (SD)	4.7 (1.9)	3.9 (1.7)	4.2 (2.1)	4.5 (2.0)	<0.0001
HUI-3, Mean (SD)	0.58 (0.23)	0.59 (0.23)	0.65 (0.21)	0.60 (0.23)	0.0163
ALHQ, Mean (SD)	2.46 (0.36)	2.28 (0.35)	2.29(0.35)	2.39(0.37)	<0.0001
Hearing loss severity					
<40 dB HL	114 (33.4)	3 (3.1)	23 (19.7)	140 (25.2)	<0.0001
≥40 dB HL	227 (66.6)	94 (96.9)	94 (80.3)	415 (80.3)	
Cogstate Brief Battery					
Detection test scores	2.60 (0.10)	2.59 (0.10)	2.56 (0.10)	2.59 (0.10)	0.0003
Identification test scores	2.77 (0.08)	2.77 (0.09)	2.76 (0.10)	2.77 (0.09)	0.2784
One-Back scores	1.35 (0.17)	1.39 (0.17)	1.39 (0.15)	1.37 (0.16)	0.0266
One Card Learning	1.03 (0.11)	1.03 (0.10)	1.05 (0.12)	1.03 (0.11)	0.2559

HUI3, Health Utilities Index Mark 3; ALHQ, Attitudes toward Loss of Hearing Questionnaire; SSQ, Speech, Spatial and Qualities of Hearing Scale.

## Results

A total of 617 participants with complete CBB or cognitive functioning data were included in the analyses: 387 hearing aid users, 103 bimodal device users, and 127 cochlear implant users. We compared study characteristics of participants with complete data with those who were excluded from analysis (*n* = 130), the only significant difference was in age i.e., participants were significantly younger compared to non-participants: 68.8 versus 73.1 years, *p* < 0.0001. Within the hearing aid group, there were 40 unilateral and 347 bilateral device users. In the cochlear implant group, there were 54 unilateral and 73 bilateral device users. Table 1 presents demographic, hearing, and psychosocial characteristics of the sample stratified by device group. Significant differences in age, HUI-3 (quality of life), ALHQ (psychosocial adjustment to hearing loss) and SSQ (self-reported hearing ability) scores were observed across the three device user groups. A higher proportion of cochlear implant and bimodal device users had moderate to severe hearing loss (≥40 dB HL) than hearing aid users (Table 1). Additionally, crude mean One Back scores were significantly lower in hearing aid users (i.e., lower accuracy or poorer working memory) compared to the other two device user groups. Conversely, hearing aid users had significantly higher mean Detection scores (i.e., slower reaction time or poorer psychomotor function) compared to the other device user groups (Table 1).

After adjusting for all potential confounding variables and conservative correction for multiple comparisons, there was limited evidence of broad cross-sectional differences in CBB test scores across the three device user groups (Table 2). Subsequently, analyses were also stratified by age (<70 and ≥70 years), to minimize the possibility that differences observed between device user groups were confounded by age-related cognitive slowing (Table 3). In participants aged <70, cochlear implant users compared to hearing aid users had better reaction time in the following test (or lower multivariate-adjusted means scores) – Detection: 2.54 versus 2.58 (*p* = 0.02). That is, cochlear implant users demonstrated better psychomotor function and visual attention compared to hearing aid users (Table 3). Conversely, Table 3 shows that in participants aged ≥70, cochlear implant users had poorer reaction time or higher multivariate-adjusted mean Identification test scores (visual attention) compared to hearing aid users: 2.83 versus 2.78 (*p* = 0.02).

**TABLE 2:** Cognitive function among the different hearing device users (*n* = 617).

	Adjusted mean Cogstate brief battery scores (standard error)^a^			
Device user groups	Working memory (One-Back test)	Psychomotor function (Detection)	Visual attention (identification)	visual learning (one card learning)
Hearing aid				
Age-sex adjusted	1.36 (0.01)	2.59 (0.01)	2.77 (0.004)	1.03 (0.01)
Multivariate adjusted	1.36 (0.01)	2.59 (0.01)	2.77 (0.01)	1.03 (0.01)
Cochlear implant				
Age-sex adjusted	1.37 (0.01)	2.58 (0.01)	2.77 (0.01)	1.05 (0.01)
Multivariate adjusted	1.37 (0.02)	2.57 (0.01)	2.77 (0.01)	1.03 (0.01)
Bimodal				
Age-sex adjusted	1.39 (0.01)	2.60 (0.01)	2.78 (0.01)	1.03 (0.01)
Multivariate adjusted	1.40 (0.02)	2.57 (0.01)	2.78 (0.01)	1.02 (0.01)
Effect sizes (95% confidence level)	0.01 (0.00–0.03)	0.04 (0.01–0.07)	0.01 (0.00–0.02)	0.004 (0.00–0.02)

^a^Adjusted for age, sex, education level, hearing loss (measured at 0.5, 1, 2 and 4 kHz), duration of device use (number of years), HUI3, Health Utilities Index Mark 3 (HUI-3) scores, total Speech, Spatial, and Qualities of Hearing Scale (SSQ) scores, and Attitudes to Loss of Hearing Questionnaire (ALHQ) vision/dexterity subscale scores.

**TABLE 3:** Cognitive function among the different hearing device users, stratified by age.

	Adjusted mean Cogstate brief battery scores (standard error)^a^			
Device users	Working memory (one-back test)	Psychomotor function (detection)	Visual attention (identification)	Visual learning (one card learning)
<70 years				
Hearing aid	1.39 (0.01)	2.58 (0.01)	2.76 (0.01)	1.04 (0.01)
Cochlear implant	1.42 (0.02)	2.54 (0.01)**^b^**	2.73 (0.01)	1.04 (0.01)
Bimodal	1.42 (0.01)	2.57 (0.01)	2.75 (0.01)	1.03 (0.01)
Effect sizes (95% confidence level)	0.005 (0.00–0.03)	0.05 (0.01–0.10)	0.03 (0.00–0.07)	0.01 (0.00–0.03)
≥70 years				
Hearing aid	1.33 (0.01)	2.62 (0.01)	2.78 (0.01)	1.03 (0.01)
Cochlear implant	1.30 (0.03)	2.60 (0.02)	2.83 (0.02)**^c^**	1.03 (0.02)
Bimodal	1.38 (0.02)	2.60 (0.01)	2.79 (0.01)	1.02 (0.02)
Effect sizes (95% confidence level)	0.01 (0.00–0.05)	0.001 (0.00–0.01)	0.03 (0.002–0.08)	0.0004 (0.00–0.01)

^a^Adjusted for sex, education level, hearing loss (measured at 0.5, 1, 2 and 4 kHz), duration of device use (number of years), HUI3, Health Utilities Index Mark 3 (HUI-3) scores, total Speech, Spatial, and Qualities of Hearing Scale (SSQ) scores, and Attitudes to Loss of Hearing Questionnaire (ALHQ) vision/dexterity subscale scores.

^b^Cochlear implant compared to hearing aid users: *p* = 0.02

^c^Cochlear implant compared to hearing aid users: *p* = 0.02

## Discussion

This study addresses a critical knowledge-gap in contemporary hearing care by evaluating whether different hearing interventions are associated with differing cognitive outcomes. Among adults aged 40+, most device-group differences in CBB test scores did not remain statistically persuasive once family-wise error was controlled; clinically, this suggests that device type alone does not reliably separate cognitive performance in this cross-sectional sample. However, age-stratified analyses revealed important heterogeneity. Among adults aged <70 years, cochlear implant users had faster reaction times on the Detection task compared with hearing aid users, indicating better psychomotor function. In contrast, among participants aged ≥70 years, cochlear implant users exhibited reduced visual attention relative to hearing aid users.

Our study suggest that device type is not uniformly associated with cognition, that is after correction, there is limited evidence for broad between-device cognitive differences at baseline. This supports prior published work which consistently suggests that cognitive outcomes in hearing loss are likely to be influenced by multiple interacting pathways (listening effort/cognitive load, sensory deprivation, and downstream effects on social participation and mental health) (Fulton et al., 2015; Griffiths et al., 2020; Livingston et al., 2024), and therefore, effects are unlikely to be identical across individuals or devices. Large-scale trials and reviews also report mixed or uncertain cognitive benefits, with signals sometimes confined to higher-risk subgroups rather than the full sample; supporting the idea that “average effects” can look null even when some patients benefit (Lin et al., 2023; Tang et al., 2025). In other words, this pattern is exactly what would be expected if cognition is not determined by the device alone, but by timing of intervention, severity/duration of loss, comorbidity profile, and baseline cognitive vulnerability, and different cognitive domains (processing speed/attention vs. learning vs. working memory) that respond differently to sensory restoration and aging.

The contrasting age-specific patterns in cognitive function observed in hearing device users in this study are particularly informative. Study findings suggest that in midlife and early older age (<70 years), cochlear implantation compared to hearing aids may help preserve processing speed. The underlying pathways for this observation are unclear. One potential mechanism is that improved hearing with cochlear implantation may facilitate greater participation in work, social interaction, and complex listening environments (Clark et al., 2012; Miller et al., 2015; Tang et al., 2024). These activities can help maintain processing speed, particularly earlier in the aging trajectory. Alternatively, studies suggest that cochlear implants can improve the timing and reliability of auditory input relative to visual cues, particularly through enhanced audiovisual integration (Rouger et al., 2007). Improved audiovisual integration may support more faster stimulus detection, which is reflected in the speed of completing cognitive tasks (Meijer et al., 2018) such as the Detection test in our study. Finally, processing speed rely on efficient frontoparietal and subcortical networks that are highly sensitive to sensory degradation. By restoring access to sound more effectively than hearing aids (Miller et al., 2015), cochlear implantation may help maintain neural efficiency and synchrony within these networks at a stage of life when plasticity is still relatively preserved (Lazard et al., 2023).

In contrast, among cochlear implant users aged ≥70 years, we observed limited cognitive gains evidenced by poorer visual attention compared to other device users. These observations are consistent with a prior study (Burkhardt et al., 2022) which reported a significant age effect across all cognitive tests in both cochlear implant users and normal hearing individuals. That is, older cochlear implant users and normal hearing individuals (60–79 years) versus younger participants (<60 years) showed poorer performance in working memory, cognitive flexibility, verbal learning and retrieval tasks. Recent research involving a large Australian dataset of 2,251 cochlear-implant recipients (Lazard et al., 2023) compared speech scores before implantation and over a 2-year period, and identified a threshold around age 70 at which performance does not increase to the same extent as the rest of the population, or may even decline. The authors attributed this finding to less robust central auditory plasticity in recipients aged over 70 (Lazard et al., 2023). This diminished auditory plasticity likely constrains the brain’s ability to efficiently integrate and automate new auditory input, which in turn maintains high cognitive processing demands and reduces transfer to attention and working memory systems; explaining the observed limited cognitive gains despite cochlear implantation. Another likely explanation is that in adults aged ≥70, cochlear implant users may exhibit slower visual attention and processing speed than the other device users because they represent a subgroup with more cumulative sensory and neurocognitive vulnerability (Burkhardt et al., 2022). Specifically, severe or long-standing hearing loss leads to increased listening effort, diverting neural resources away from attention. While cochlear implants improve hearing acuity, they may not fully reverse decades of sensory deprivation or central auditory reorganization, particularly in advanced age. Nevertheless, the cross-sectional design precludes concluding cochlear implants cause poorer attention, rather it flags the need in cochlear implant users aged ≥70 for targeted monitoring of attention/processing-speed-related difficulties during rehabilitation and follow-up because slower visual attention could affect listening effort, adherence, and functional communication outcomes.

Moreover, in interpreting these results, it is important to consider effect sizes in addition to statistical significance. In a cohort of this size, small between-group differences can yield statistically significant *p*-values; however, the magnitude of observed effects indicates that differences observed in age-stratified analyses were relatively modest. Across outcomes, the partial eta-squared estimates were generally in the small-to-moderate range (0.01–0.06), indicating that hearing device group accounted for only a limited proportion of variance in CBB test scores. Therefore, while the direction of findings is clinically informative, these differences should not be interpreted as determinative at the individual patient level. Rather, they highlight group-level patterns that may help clinicians identify domains (e.g., processing speed and visual attention) where additional support is needed.

The public health and clinical implications of this study are numerous. First, this study provides evidence that different hearing interventions are associated with different cognitive outcomes, particularly in domains central to functional independence (attention and processing speed). This reinforces the importance of hearing loss management in minimizing cognitive decline, supporting its inclusion in dementia-prevention and healthy aging strategies and policies. Second, the age-specific patterns suggest a critical window before 70 years of age during which cochlear implantation may better preserve processing speed and attentional efficiency. That is, promoting timely intervention may maximize both sensory and cognitive returns in adults with severe to profound hearing loss. This has important implications for public health messaging around earlier hearing loss detection and referral. Third, the observed heterogeneity across age and device type highlights the need for stratified hearing services. Hearing healthcare should move beyond device provision alone to incorporate age, cognitive risk, and functional goals into care planning.

A major strength of this study is the analysis of a relatively large and diverse sample of hearing device users, and the comprehensive collection of hearing and health data. Further, the use of the CBB enabled the visual presentation of cognitive assessments, therefore, reducing confounding of cognitive data by hearing loss. However, this study is not without limitations. The cross-sectional study design precludes causal inference; temporal data are required to evaluate the direction of observed associations and to determine whether device-related improvements in cognitive function are sustained over time. Additionally, while we adjusted for a range of potential covariates, there may be residual confounding from covariates that were not considered or measured (e.g., health seeking behaviors, social isolation and physical activity). Finally, our findings need to be interpreted with caution as we did not have appropriate comparator groups i.e., participants with hearing loss but not using a hearing aid and participants with no hearing loss.

In summary, these findings advance the field by demonstrating that the relationship between hearing intervention and cognition is age-dependent, and domain- and device-specific. They support a shift toward more targeted hearing care, where the timing and type of intervention are tailored not only to auditory needs, but also to cognitive health and aging trajectories. This research also strengthens the case for earlier and person-centered hearing rehabilitation as a strategy to support healthy aging and long-term brain health in adults with hearing loss.

## Data Availability

The raw data supporting the conclusions of this article will be made available by the authors, without undue reservation.
